# Structural Analysis of the Wheat Genes Encoding NADH-Dependent Glutamine-2-oxoglutarate Amidotransferases and Correlation with Grain Protein Content

**DOI:** 10.1371/journal.pone.0073751

**Published:** 2013-09-17

**Authors:** Domenica Nigro, Yong Q. Gu, Naxin Huo, Ilaria Marcotuli, Antonio Blanco, Agata Gadaleta, Olin D. Anderson

**Affiliations:** 1 Department of Soil, Plant and Food Sciences, Section of Genetic and Plant Breeding, University of Bari “Aldo Moro”, Bari, Italy; 2 Genomics and Gene Discovery Research Unit, Western Regional Research Center, USDA-ARS, Albany, California, United States of America; Ben-Gurion University, Israel

## Abstract

**Background:**

Nitrogen uptake and the efficient absorption and metabolism of nitrogen are essential elements in attempts to breed improved cereal cultivars for grain or silage production. One of the enzymes related to nitrogen metabolism is glutamine-2-oxoglutarate amidotransferase (GOGAT). Together with glutamine synthetase (GS), GOGAT maintains the flow of nitrogen from NH_4_^+^ into glutamine and glutamate, which are then used for several aminotransferase reactions during amino acid synthesis.

**Results:**

The aim of the present work was to identify and analyse the structure of wheat *NADH-GOGAT* genomic sequences, and study the expression in two durum wheat cultivars characterized by low and high kernel protein content. The genomic sequences of the three homoeologous A, B and D *NADH-GOGAT* genes were obtained for hexaploid *Triticum aestivum* and the tetraploid A and B genes of *Triticum turgidum ssp. durum*. Analysis of the gene sequences indicates that all wheat *NADH-GOGAT* genes are composed of 22 exons and 21 introns. The three hexaploid wheat homoeologous genes have high conservation of sequence except intron 13 which shows differences in both length and sequence. A comparative analysis of sequences among di- and mono-cotyledonous plants shows both regions of high conservation and of divergence. qRT-PCR performed with the two durum wheat cvs Svevo and Ciccio (characterized by high and low protein content, respectively) indicates different expression levels of the two *NADH-GOGAT-3A* and *NADH-GOGAT-3B* genes.

**Conclusion:**

The three hexaploid wheat homoeologous *NADH-GOGAT* gene sequences are highly conserved – consistent with the key metabolic role of this gene. However, the dicot and monocot amino acid sequences show distinctive patterns, particularly in the transit peptide, the exon 16–17 junction, and the C-terminus. The lack of conservation in the transit peptide may indicate subcellular differences between the two plant divisions - while the sequence conservation within enzyme functional domains remains high. Higher expression levels of *NADH-GOGAT* are associated with higher grain protein content in two durum wheats.

## Introduction

Nitrogen uptake is an essential element in crop improvement, either directly for grain protein content or indirectly for photosynthetic production. Thus, nitrogen utilization is fundamental to crop productivity. Over the past 50 years nitrogen (N) fertilizers have been extensively used to increase both grain yield (GY) and grain protein content (GPC) in cereals - helping to support a vastly increased world population. Despite that GY and GPC are genetically negatively correlated, it has been shown in various cereals (including wheat) that this correlation can be broken down by adequate nitrogen supply in late plant development [1], [2]. However, this requires that growers must optimize the use of nitrogen fertilizers to avoid pollution, while maintaining reasonable profit margins. Improved crops should make better use of nitrogen fertilizer supplies giving higher yields with improved protein contents. Therefore, selecting new crop varieties exhibiting improved nitrogen use efficiency (NUE; the yield of grain per unit of available nitrogen in the soil), and adapting agricultural practices to reduce the use of nitrogen fertilizers represent challenges for both breeders and farmers [3].

Whether nitrogen is derived from soil reserves, from nitrogen fertilizer, or from N_2_ fixation, it is incorporated into the organic form via the assimilation of ammonia. However, the primary assimilation of ammonia from external inorganic nitrogen is only part of the process. Nitrogen is also released from plant metabolism as ammonia and reassimilated many times during the movement of nitrogen around the plant, from seed reserve, through transport to vegetative organs, to eventual redeposition in a new crop of seeds. There is also a major release and reassimilation of nitrogen during the process of photorespiration in C3 plants. The process of ammonia assimilation is thus of crucial importance to crop growth and productivity.

Two major enzymes are responsible for cyclic assimilation of ammonium into amino acids in the biochemical pathway of NH_4_^+^ assimilation; i.e., glutamine synthetase (GS) and glutamine-2-oxoglutarate amidotransferase (GOGAT). These two enzymes are involved in assimilation and recycling of mineral nitrogen catalyzing ATP-dependent conversion of glutamine into glutamate using ammonia as substrate [4], [5]. Glutamine synthetase exists in multiple enzyme forms, the chloroplastic isozyme being encoded by one gene (*GS2*) and the cytosolic form organized in sub-family of genes. Studies have shown that both chloroplastic and cytosolic GS isozymes are developmentally regulated in leaves [6], [7], [8], [9] and have different metabolic roles. The GOGAT enzyme catalyzes the reductive transfer of the amide group of glutamine to 2-oxoglutarate to form two glutamate molecules [1]. Together with GS, it maintains the flow of nitrogen from NH_4_^+^ into glutamine and glutamate, which are then used for several other aminotransferase reactions during the synthesis of amino acids [5]. Kinetic and inhibitory studies have suggested that GOGAT is the rate-limiting step in amino acid production [10], [11]. In rice, two different GOGAT enzymes have been identified based on the electron donor; i.e., a ferredoxin (Fd)-dependent GOGAT and a NADH-dependent GOGAT. Also in rice, NADH-GOGAT is active in developing organs, such as unexpanded non-green leaves and developing grains [12]. NADH-GOGAT has been proposed to be involved in the use of remobilized nitrogen, because it is located in the specific cell types which are important for solute transport from the phloem and xylem elements [13].

A recent report showed, through cereal genome paleo-history studies, that a NUE QTL has been conserved at the same orthologous loci as the *GOGAT* gene on wheat chromosome 3B, rice chromosome 1, sorghum chromosome 3 and maize chromosomes 3 and 8, despite 50–70 million years of separate evolution associated with considerable sequence shuffling (50 rearrangement events within the NUE locus region) [14]. For these reasons, *NADH-GOGAT* is one of the potential candidate genes involved in the control of the complex character trait GPC.

The aims of the present work were the identification of wheat *NADH-GOGAT* A, B and D genomic sequences, the determination of their structure, a comparison to GOGAT sequences to other plants, and the study of *NADH-GOGAT* expression in two durum wheat cvs characterized by low and high GPC.

## Results

### Assembly of Hexaploid and Tetraploid *NADH-GOGAT* Gene Sequences

A previously reported rice genomic sequence of *NADH*-*GOGATI* [15] was used to extract 487 454-sequences of hexaploid wheat (A-, B-, and D-genomes) cv Chinese Spring (cerealsdb.uk.net/search_reads.htm) and 82 454-reads of the D-genome from the diploid *Triticum tauschii* (avena.pw.usda.gov/RHmapping/blast/). These two groups of 454 reads were separately assembled. The Chinese Spring assembly produced a single contig comprised of the three distinct sequences. By comparing this contig with the reported chromosome 3B genomic sequence containing a *NADH-GOGAT* gene (FN564429) and the sequence obtained by assembling D-genome 454-sequences, we were able to separate the 487 Chinese Spring 454-reads into three sequences assignable to each of the three genomes: 178 reads to the A-genome, 162 to the B-genome, and 147 to the D-genome (the A-genome assignment was by elimination). The three hexaploid wheat homoeologous *NADH-GOGAT* gene sequences are given in File S1.

**Figure 1:**
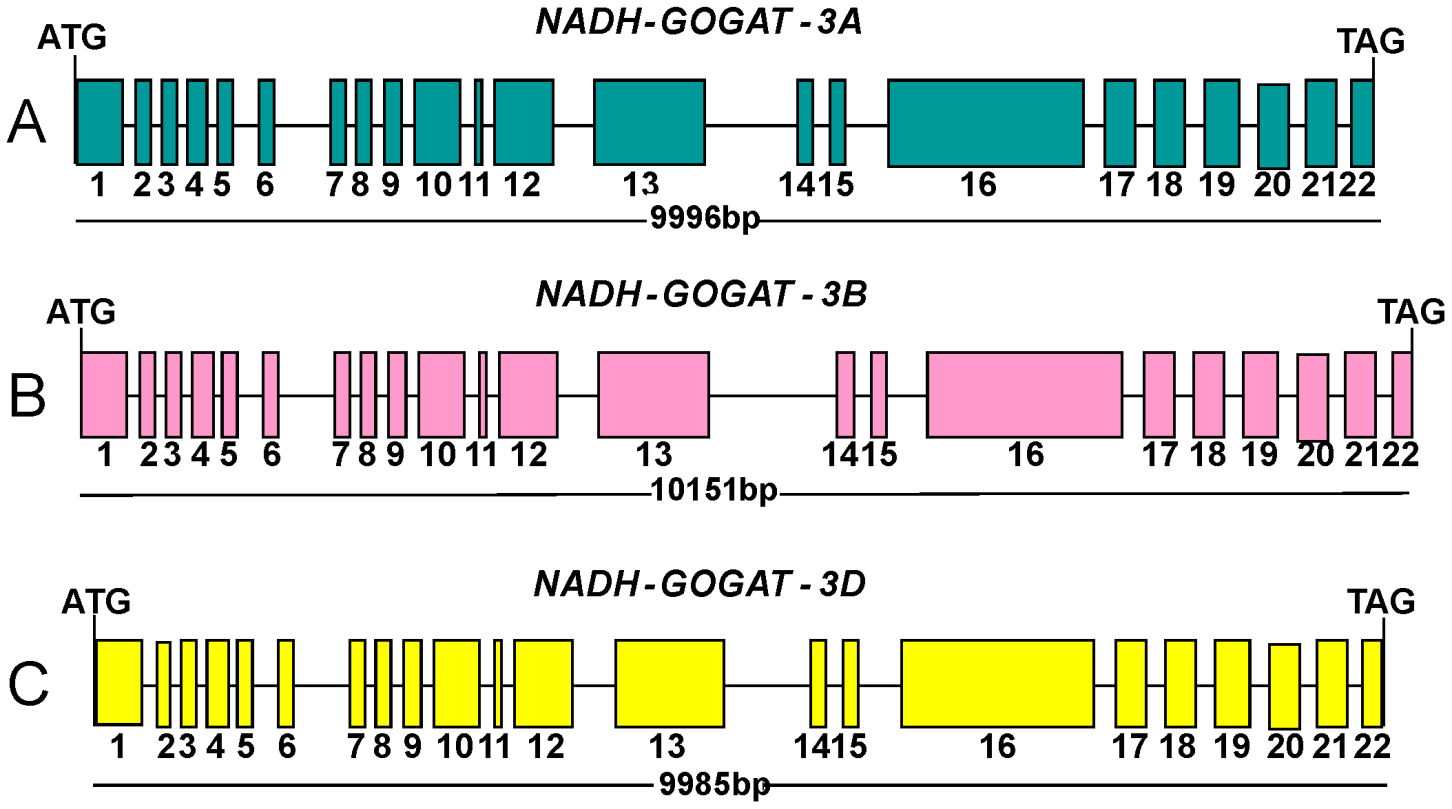
Exon/intron organization of wheat *NADH-GOGAT* genes. A. A genome *NADH-GOGAT*, B. B genome *NADH-GOGAT* and C. D genome *NADH-GOGAT*. Exons are indicated by numbered boxes and introns by intervening lines. Start and stop codon positions of the full-length coding regions are marked.

Analysis of the sequences finds that the wheat *NADH-GOGAT* gene is comprised of 22 exons and 21 introns for all the three homoeologous genes (Figure 1). The previously reported sequence for rice showed one more 5′ exon entirely within the 5′ UTR and is not translated into protein. The rest of the rice gene shows a very high similarity in exon/intron length and organization with the three genes derived for wheat. The intron borders matched the canonical plant intron borders (GT…AG) for all 21 introns. Consensus exon/introns boundaries were determined using grass ESTs sequences aligned with the genomic sequence. When there were no overlapping wheat EST sequences, as happened for the first 5 kb at the 5′ end of the genomic sequence, we used other grasses EST sequences to determine the boundaries. In regions with few or no wheat ESTs, exon/intron boundaries matched rice and maize sequences in all cases.

Although the three genes show the same exon/intron number and size, an exception is intron 13, which shows major differences in both length and sequence for all of three homoeologues (Figure S1).

Since there is neither information about wheat *NADH-GOGAT* DNA coding nor amino acidic sequences, the precise the 5′ UTR start is uncertain. However, from the starting codon ATG to the stop codon TAG the three genes show a similar length: the *NADH-GOGAT-3B* gene is the longest at 10,151 bp, the *NADH-GOGAT-3A* is 9,996 bp long, and the gene located on chromosome 3D is the shortest at 9,985 bp. (Figure 1).

The genomic sequence and structure of the 3A and 3B *NADH-GOGAT* genes were also obtained for the two durum wheat cultivars Svevo and Ciccio using primers designed from the Chinese Spring sequences. No polymorphisms were found for either gene between the two durum varieties. The only differences between the hexaploid and tetraploid wheats are few single nucleotide polymorphisms and single nucleotide indels, all located in introns. The identical tetraploid Svevo and Ciccio DNA sequences are available at NCBI under accessions KC960545 for the 3A gene and KC960544 for the 3B gene.

### Comparison of the Three Hexaploid *NADH GOGAT* Homoeologous Genes

**Figure 2:**
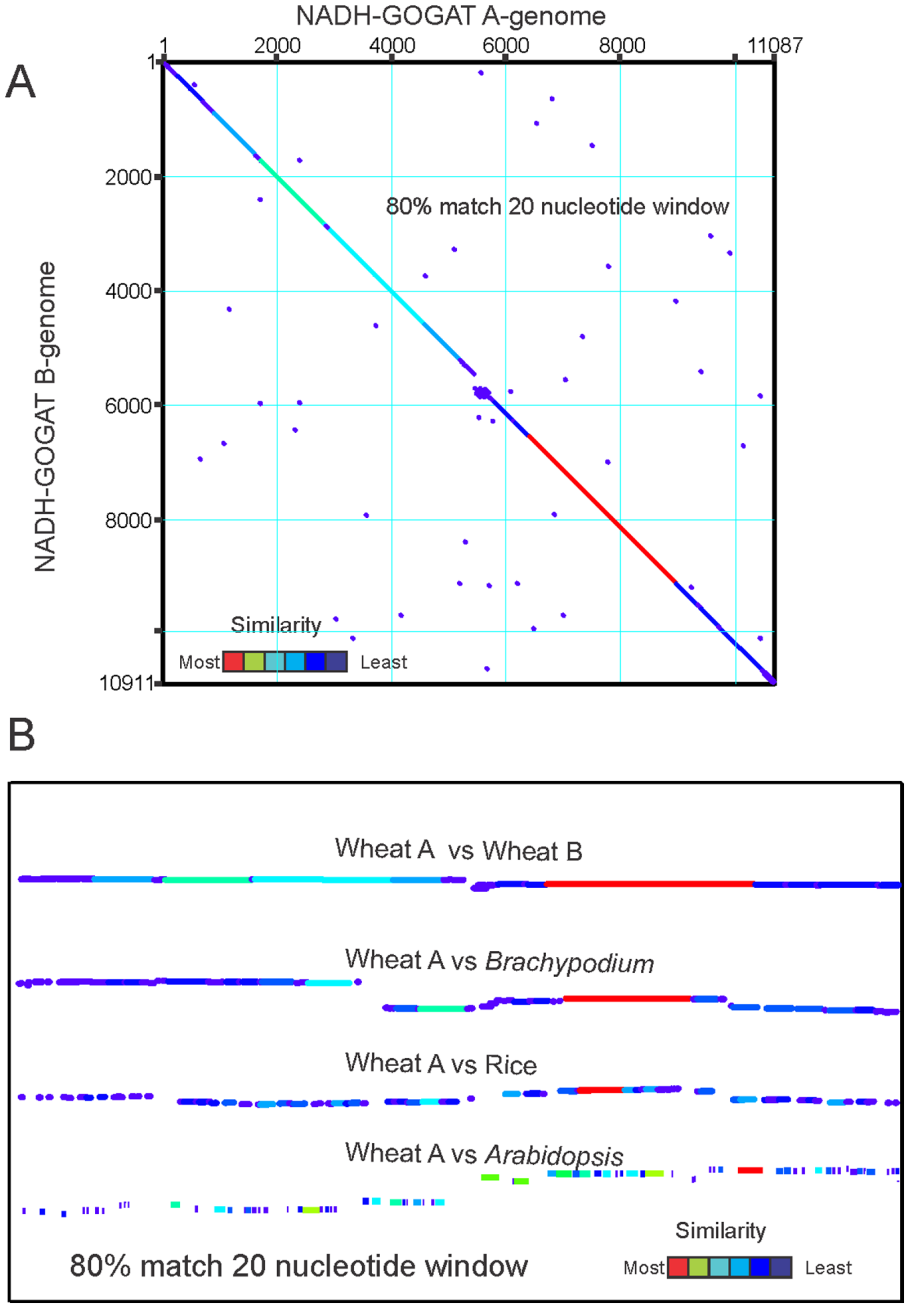
GOGAT sequence comparisons. *NADH-GOGAT* DNA sequences from start to stop codons, including both exons and introns are compared to show relative conservation of sequences. A. Sequences of *THE NADH-GOGAT* genes from the A- and B-genomes. Match criterion of 80% match over a 20 residue window. B. DNA sequences from start to stop codons were compared between indicated plants using a criterion on 80% match over a 20 base window. Dot blot diagonals are arrayed horizontally for comparison. In both frames the color-coded heat map represents a composite of the degree and length of sequence similarity.

The three hexaploid *NADH-GOGAT* genes are highly conserved as seen in the dot plot example of the entire A-genome GOGAT vs the B-genome GOGAT genes using the criterion of an 80% match over a 20 base window (Figure 2A). The conservation extends extensively into the introns as seen by increasing the match criterion to 100% over a 20 base window (Figure S2). Only intron 13 shows both major sequence and length differences. The other pairwise comparisons (A vs D, B vs D) show the same pattern (not shown).

Focusing on exon sequences only, the alignments between two homoeologues at a time showed a total of 84 SNPs spread all over the 22 exons in the *NADH-GOGAT*-*3A* and *NADH-GOGAT-3B* comparison. Fewer SNPs are found in the *NADH-GOGAT-3A* and *NADH-GOGAT*-*3D* comparison (71 SNPs) or the *NADH-GOGAT-3B* and *NADH-GOGAT*-*3D* comparison (67 SNPs). The same situation is found when only introns sequences are compared: *NADH-GOGAT-3A* and *NADH-GOGAT-3B* showed a total of 73 SNPs spread all over the 20 introns (intron 13 excepted), with *NADH-GOGAT-3A* and *NADH-GOGAT-3D* introns different in the same regions for 67 SNPs, *NADH-GOGAT-3B* and *NADH-GOGAT-3D* introns different by only 48 SNPs. In addition, the three pairwise comparisons find intron sequence differences to include 17 short indels (Figure S1). Intron 13 is the longest intron at 706, 831, and 688 bases for the A, B, and D genomes, respectively - with the central approximately 300 bp of the three sequences differing among the three homoeologues (Figure S1).

**Figure 3:**
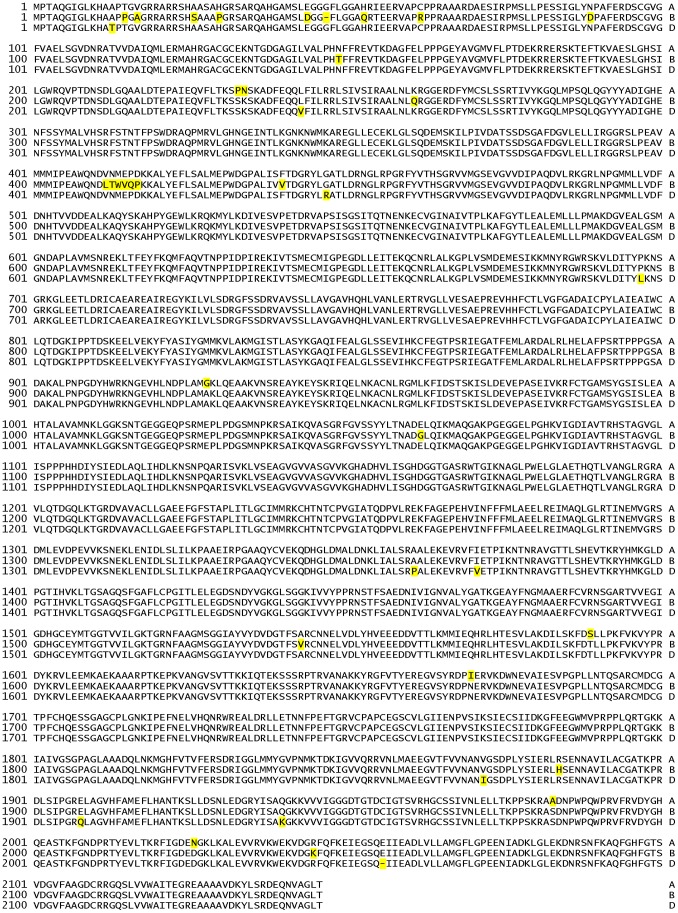
Comparison of three hexaploid NADH-GOGAT amino acid sequences. The three cv Chinese Spring NADH-GOGAT polypeptide sequences are aligned. Genomes are indicated on the right by letter of the genome (A, B, D). Differences in amino acid residues are indicated by yellow shading.

A high conservation is also seen in the derived amino acid sequences as shown aligned in Figure 3. The are 41 total sequence differences among the three homoeologues –39 residue changes and two fewer amino acids. The latter occurs in the transit peptide portion of *NADH-GOGAT-3B* and near the C-terminal end of *NADH-GOGAT-3D*. Of the 39 single residue changes in the 3-way comparison of Fig. 3, 22 occur in the B-genome sequence compared to 7 and 10 differences in the A- and D-genome proteins, respectively.

### Comparison of Amino Acid Sequences from Other Species

**Figure 4:**
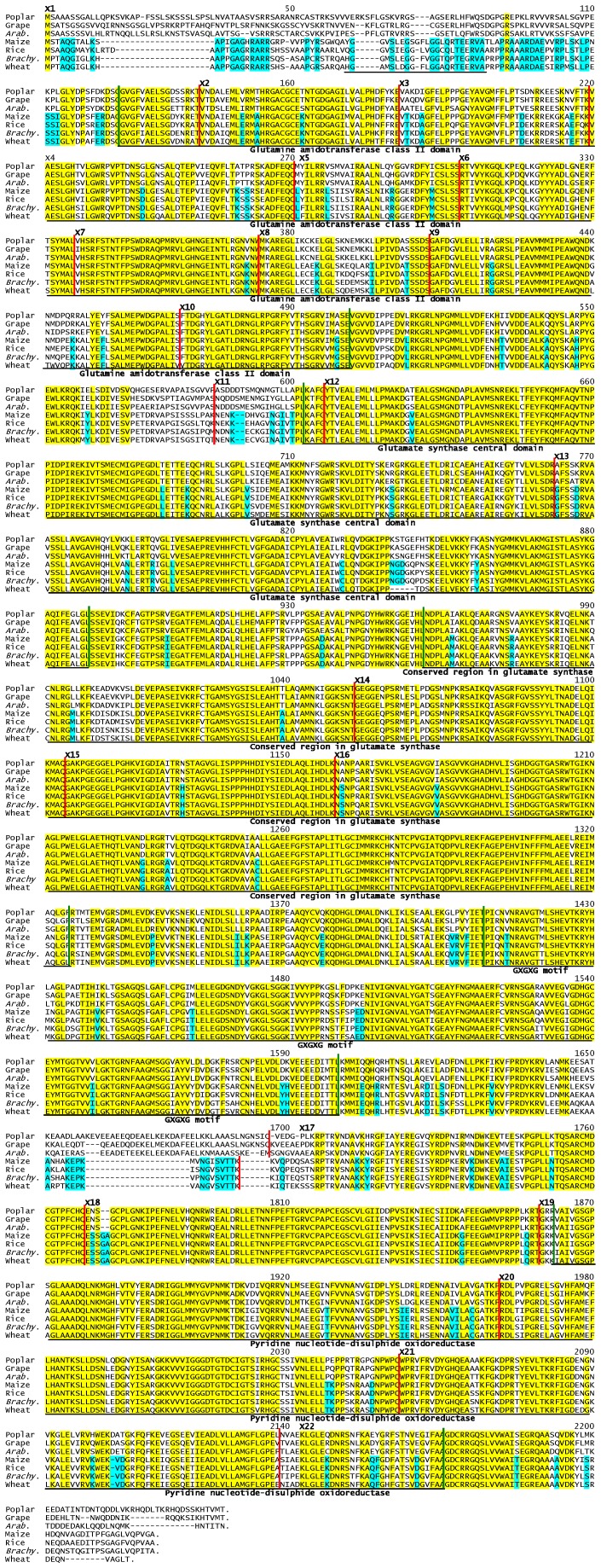
Dicot vs Moncot NADH-GOGAT amino acid sequences. The derived amino acid sequence of the wheat B-genome NADH-GOGAT protein is compared to sequences derived from DNA sequences of other plants (poplar, grape, *Arabidopsis* (*Arab.*), maize, rice, and *Brachypodium* (*Brachy.*). Amino acid residues conserved in all aligned proteins are shaded in yellow. Amino acids unique to monocots are shaded blue. Vertical red lines indicate exon boundaries. Exon numbers, such as ‘X5’ for exon 5, are to the right of the exon boundary lines. Known or presumptive functional domains of the NADH-GOGAT enzyme are below the alignment – with approximate domain boundaries [16] indicated by vertical green lines.

The degree of conservation of amino acid sequences was shown among a selection of plant genomic sequences. The coding sequence from the wheat *NADH-GOGAT-3B* gene was used to derive the complete 3B amino acid sequence, and a similar analysis was carried out for available sequences from *Brachypodium*, grape, poplar, soybean, *Arabidopsis* and rice. The seven derived amino acid sequences are aligned in Figure 4. The wheat exon-encoding boundaries are indicated by vertical red lines. There is an additional 5′ exon in rice and grape that is entirely 5′ UTR sequence; e.g., exon 2 in wheat would be exon 3 in rice and grape.

Highlighted in yellow are conserved amino acid positions on all analyzed plants and positions unique to monocots are highlighted in blue. Exon encoded positions are conserved among all plants analyzed with the previously mentioned exception for the first exon and the exon 16–17 junction point in comparing dicot and monocot plants. Although much of the sequence is conserved among all plants, there is a distinctive pattern of sequence differences between the dicots and monocots as seen in the blue highlighting of Figure 4. In addition, several sequence regions are characterized by both residue differences and sequence length variation. The major examples of differences between monocots and dicots include the junction of exons 16 and 17, and the beginning and ends of the polypeptides.

The only other notable difference in the wheat sequences are deletions of five and eight amino acid residues at positions 840 and 2205, respectively, in Figure 4. The latter deletion was reconfirmed by rechecking the 454 reads and with wheat ESTs, and the former confirmed only with the 454 reads as there are no Triticeae ESTs in that region. Both sequence indels occur within non-conserved regions.

The conservation of DNA sequence extends into the intron sequences, but this conservation decays with evolutionary distance as seen in Figure 2B where the diagonals of four dot blots are array horizontally for comparison. The diagonals compare the gene sequences (from start to stop codons) of the 3A vs 3B genes shown in Figure 2A and the wheat 3A gene vs *NADH-GOGAT* genes of *Brachypodium*, rice, and *Arabidopsis* (Figure 2B). The *Brachypodium* result shows the similarity to wheat extends extensively into the introns, whereas the matching sequence in rice is largely exonic with limited intron similarity. By the evolutionary distance represented by *Arabidopsis*, the sequence similarity is restricted to exons and short sequences at the intron ends.

Below the alignment of the NADH-GOGAT enzymes in Figure 4 are noted the four known functional domains – indicated by names, lines, and green vertical lines at the domain ends [16].

### Chromosome Mapping of the NADH-GOGAT Genes

Since the genes in the two durum cultivars (Ciccio and Svevo) had no SNPs, the Ciccio/Svevo mapping population would not be used to map the chromosome positions. However, the positions in the hexaploid cv Chinese Spring were mapped using genetic stocks including nulli-tetrasomic, di-telosomic and a set of wheat deletions bin lines (described in Material and Methods).

**Figure 5:**
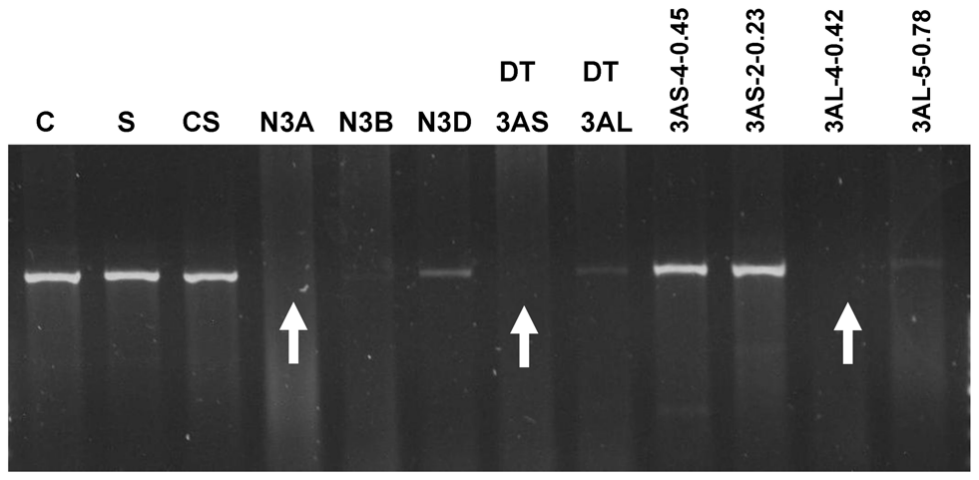
Physical mapping of the *NADH-GOGAT* gene on chromosome 3A. A gene specific marker was amplified in cvs Ciccio (C), Svevo (S), Chinese Spring (CS) and a set of nullitetrasomic and bin deletion lines. The 1 kb fragment was absent in the nulli-3A-tetra-3D line, in the ditelo-3AS, and in the bin line 3AL-4-0.42, as indicated by arrows.

*NADH-GOGAT-3A* and *NADH-GOGAT-3B* genes were physical located to the 3A and 3B chromosomes, respectively, using nulli-tetrasomic lines of cv Chinese Spring (Figure 5) – then further localized to the long arms of those chromosomes (using ditelocentric 3AS lines missing the long arms) and then to bins 3AL-0.42-0.78 and C-3BL2-0.50. Only the 3A results are shown in Figure 5 for the arm and bin mappings.

### Expression Profile of NADH-GOGAT Genes in Wheat

General patterns of *NADH-GOGAT* gene expression were determined by two approaches. The first approach was to estimate if there was differential expression in hexaploid wheat among the three homoeologous genes. To determine this, 67 wheat ESTs encoding wheat *NADH-GOGAT* sequences were identified at NCBI. When matched to the three wheat homoeologous *NADH-GOGAT* sequences, 23 match to the 3A gene, 19 to 3B, and 15 to 3D. A Chi-square test for random distribution of the ESTs results in p = 0.43, indicating the distribution provides no support for preferential distribution and therefore no support for differential gene expression.

**Figure 6:**
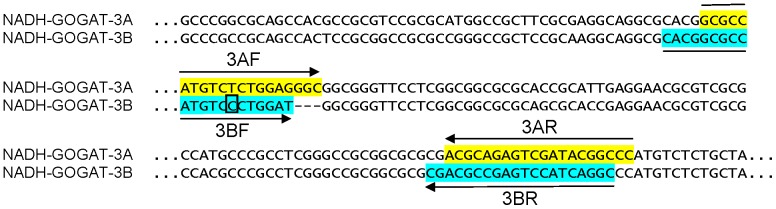
*NADH-GOGAT* primers. *NADH-GOGAT-3A* and *NADH-GOGAT-3B* specific primer combinations used in qRT-PCR analysis. The boxed base indicates the change in the *NADH-GOGAT-3B* forward primer to increase specificity. Primers are listed in Table 2.

**Figure 7:**
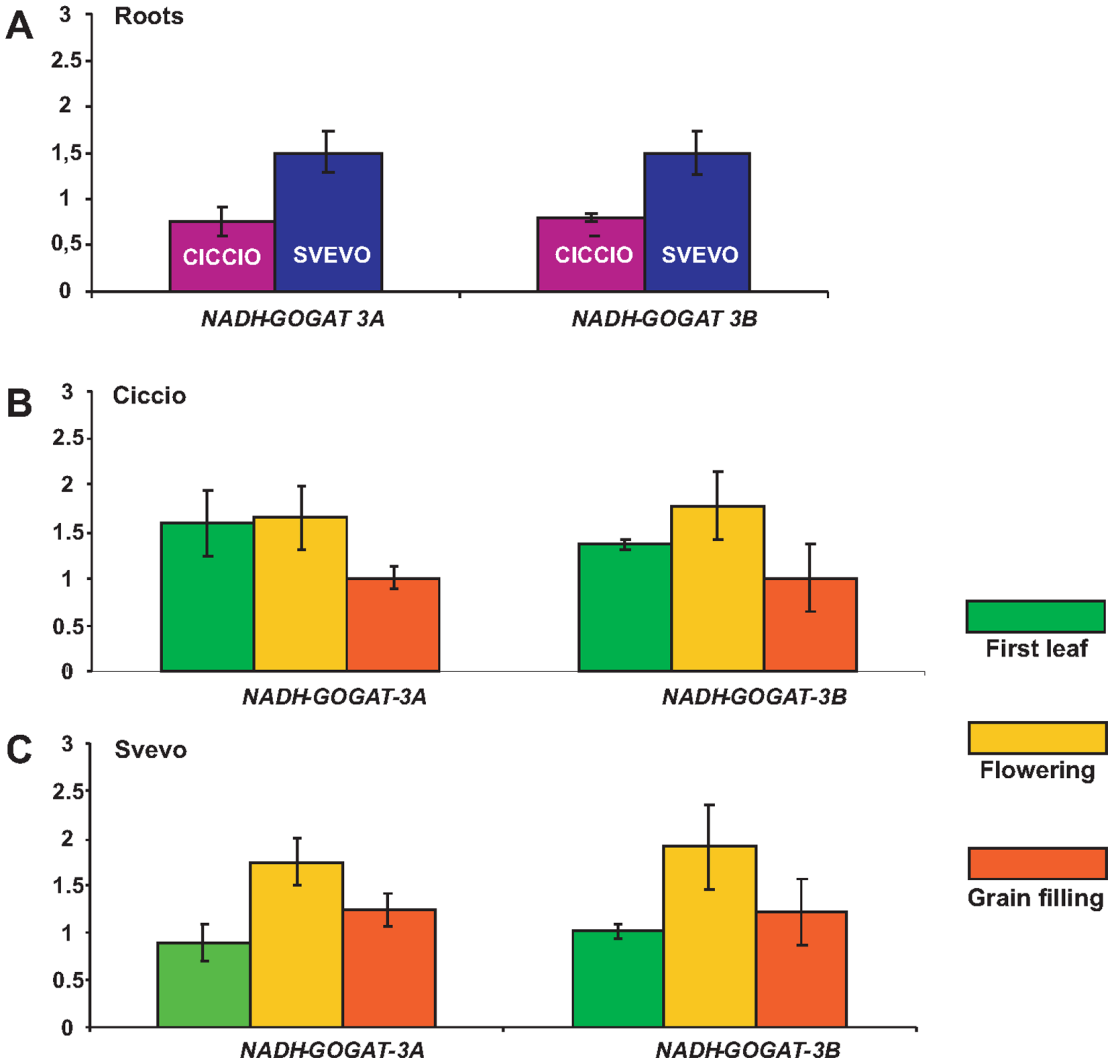
qRT-PCR of *NADH-GOGAT*. qRT-PCR conducted for *NADH-GOGAT-3A* and *NADH-GOGAT-3B* genes with specific probes. The figure shows: A. The expression levels of the two genes in the roots of the two durum wheat cvs Ciccio and Svevo (C and S, respectively); B. Comparison of the two homoeologous gene expressions in cv Ciccio during the stage of first leaf, flowering and grain filling; D. Comparison of the two homoeologous gene expressions in cv Svevo during the stage of first leaf, flowering and grain filling;. The error bars indicate the ± SE of the mean.

The second approach to examine expression was to determine *NADH-GOGAT-3A* and *NADH-GOGAT-3B* genes expression levels of the two durum wheat cvs Svevo and Ciccio from roots (collected at seedling stage) and leaves at three different phenotypic stages (first leaf, flowering and grain filling). Total RNA was extracted from tissues of plants grown in field conditions and reverse-transcribed for qRT-PCR analyses. To test if the homoeologous genes show differential expression patterns, qRT-PCR were performed using specific primers designed to preferentially amplify the A and B sequences (Figure 6). In roots, significant differences (P<0.01) were observed in the expression of both homoeologous tetraploid genes (*NADH-GOGAT-3A* and *NADH-GOGAT-3B*) between the two cultivars Ciccio and Svevo, characterized by different grain protein content values (about 14% and 15%, respectively [17]). Roots of cv Svevo, the durum cultivar with the higher final kernel protein content [17], showed the highest level of both genes transcript (Figure 7A). In particular, the relative ratio of *NADH-GOGAT-3A* and *NADH-GOGAT-3B* transcript abundances were both 1.50 in cv Svevo, while the transcript abundances were 0.75 and 0.80 in cv Ciccio, for A and B homoeologous, respectively.

In leaves, transcript levels in the first leaf and grain filling stages showed similar expression levels, while a significantly higher value of transcripts was observed during flowering (P<0.01). *NADH-GOGAT-3A* and *NADH-GOGAT-3B* relative mRNA abundances at flowering were 1.78 and 1.00, respectively, in cv Ciccio, and 1.71 and 1.91, respectively, in cv Svevo.

A similar trend was observed for both the homoeologous genes and in both cultivars (Figure 7B–C). Statistical analysis of all data with ANOVA did not show significant differences among biological and technical replicas demonstrating a stability of genes expression even in field conditions. In cv Svevo, a higher level of the *NADH-GOGAT-3B* transcript was found compared to *NADH-GOGAT-3B* of the cv Ciccio. These results are in accordance with the fact that several papers have reported a QTL for GPC on 3B chromosome in the same bin in which the gene is located [14], [17]. No significant differences were observed in the expression of the *NADH-GOGAT-3A* in leaves between the two cultivars.

## Discussion

The current report describes the determination of the wheat *NADH-GOGAT* gene sequences in hexaploid and tetraploid wheat. This gene is one of the candidate genes involved in the control of the complex character grain protein content (GPC). The recent and constant increase in partial or complete genome sequence data opens up interesting perspectives for the use of such large data resources. Through comparative genomics and using new generation sequencing data, it will be easier to obtain information about metabolisms and/or genes of interest in very large and high repetitive genomes such as wheat. Positional cloning of genes from the numerous sequenced genomes in a so-called ‘cross-genome map-based cloning’ is also a useful tool to gain information in lesser known genomes. In this study, we used the single reported sequence for rice [15] to extract from public websites all Roche 454 sequences of wheat cv Chinese Spring (http://www.cerealsdb.uk.net/search_reads.htm) and 454 reads of *Triticum tauschii* accession (http://avena.pw.usda.gov/RHmapping/blast/), the diploid ancestor of the D-genome of hexaploid wheat (ABD). The latter data was generated in our laboratory. We were able to assemble the three complete orthologous genes for the three hexaploid wheat genomes and a partial sequence from *T. tauschii*. PCR primer pairs were designed and used to identify genome assignments using DNA from Chinese Spring nulli-tetrasomic, ditelosomic, and bin-deletion lines - resulting in assigning the *NADH-GOGAT* genes to the group 3 chromosome short arm. The three homoeologous genes have the same intron/exon structure with similar SNP differences in both introns and exons. The conservation in sequence is especially seen in the amino acid sequences, which even when compared to other plants show regions of high conservation – presumably related to regions of critical functionality essential for plant metabolism.

Interestingly, there is major conservation even with intron sequences compared to more divergence of introns in another nitrogen-metabolism related gene, lysine ketoglutarate reductase saccharopine dehydrogenase (LKR-SDH) [18]. Both genes contain a large centrally located intron with the least sequence conservation. In the LKR-SDH gene, this region is referred to as a ‘hinge’ region separating the two major functional domains of the enzyme, LKR and SDH. Neither is known the exact role of this region, nor if it is also involved in separating functional domains in the NADH-GOGAT enzymes.

The conservation among NADH-GOGAT sequences can also be seen when comparing amino acid sequences. The coding sequence from the wheat *NADH-GOGAT* genes (encoded sequences indicated in Figure 4) were used to derive the complete amino acid sequences and compared with model and crop species such as *Brachypodium*, grape, poplar, soybean, *Arabidopsis* and rice. Despite the presence of residue differences among species, what is clear is that some regions of amino acid sequence are highly conserved among the species examined, most of all in the regions corresponding to the enzyme functional domains. Five different protein domain are present in NADH-GOGAT, which are in order: 1) a glutamine amidotransferases class-II (376 aa); 2) a glutamate synthase central domain (288 aa) which connects the amino terminal amidotransferase domain with the FMN-binding domain and has an alpha/beta overall topology; 3) a conserved region containing a putative FMN binding site and Fe-S cluster (369 aa); 4) a GXGXG motif, founded only in glutamate synthase and few other enzymes, and characterized by a repeated G-XX-G-XXX-G motif (192 aa); 5) and a pyridine nucleotide-disulphide oxidoreductase domain, which is actually a small NADH binding domain within a larger FAD binding domain (314 aa) [16]. Length and position of all of these domains are conserved in all the species examined, as shown in Figure 4. The wheat *NADH-GOGAT* sequence shows a higher similarity with the *Brachypodium* gene sequence in pairwise alignments and phylogenetic trees generated from multiple species alignments (not shown). This is consistent with the finding of the close sequence relationship between *Brachypodium* and the Triticeae species [19].

The comparison of this set of plant *NADH-GOGAT* genes (wheat, *Brachypodium*, rice, sorghum, poplar, *Arabidopsis*, and grape) suggests regions of greater sequence and structure conservation likely related to critical enzymatic functions and metabolic control. The three main differences in amino acid sequence among the aligned proteins in Figure 4 are the N- and C-termini, and the junction between sequences encoded by exons 16 and 17. The N-termini of NADH-GOGAT proteins is reported to encode an approximately 100 bp presequence/transit/targeting peptide in both rice [15] and bean [17]. In contrast, the alignment of Figure 4 shows that whatever the exact structure of this ‘presequence,’ there must be an approximately 25 amino acid residue difference between the aligned dicot and monocot polypeptides. In addition, the sequences in this region are also very different between monocots and dicots. It is not yet determined if these variations in length and sequence have any different functional metabolic roles in the two plant branches. Similar uncertainty exists as to whether the dicot/monocot differences in NADH-GOGAT structure at the C-terminus and junction of exon 16 and 17 encoded amino acid sequences have functional significance or are more random drift of relatively non-functional sequences since no role in metabolism is yet assigned.

Together with GS, GOGAT maintains the flow of nitrogen from NH_4_+ into glutamine and glutamate, which are then used for several other aminotransferase reactions during the synthesis of amino acids [5]. Kinetic and inhibitory studies have suggested that *NADH-GOGAT* is the rate-limiting step in amino acid production [10], [11].

A recent cross-genome ortho-meta QTL study of NUE in cereals identified a GOGAT gene as a major candidate for cereal NUE [14]. In rice, the suppression of both GOGAT genes reduced yield per plant and thousand kernel weight, phenotypic indications of nitrogen starvation [20]. Transgenic wheat cv Kasalath lines over-expressing the NADH-GOGAT rice orthologue (accession no. AB001916 corresponding to LOC_Os01g48960) under the control of its own promoter showed an increase in grain weight (80% maximum), supporting that NADH-GOGAT is indeed a key step in nitrogen utilization and grain filling in rice [12]. In the present study, the transcriptional level of the *NADH-GOGAT-3A* and *NADH-GOGAT-3B* genes was assessed in two durum wheat cultivars Svevo and Ciccio previously analyzed for grain protein content in several years and environmental conditions [17] and characterized by a high and low final grain protein content. qRT-PCR conducted with specific primers in order to analyze individually the two homoeologous genes revealed a differential pattern for the two genes in the two cultivars. A significantly different expression was observed between the two cultivars in roots with an higher level of transcript in the cv Svevo for both genes. This results suggest that the *NADH-GOGAT* genes play a crucial role in differencing the final grain protein content since these genes are principally active in non-photosynthetic germinating seed, roots and etiolated shoots [21].

Results indicate that, in roots, the alleles encoded by cv Svevo are more transcribed than those of cv Ciccio. In leaves, the two cultivars reverse patterns of NADH-GOGAT transcript titers, both cultivars with peaks at the flowering stage and lowest titers at first leaf stage for Svevo and grain filling stage for Ciccio. These patterns are similar for both the 3A and 3B *NADH-GOGAT* genes, and the higher titers for Svevo at grain filling is consistent with the higher Svevo titers in roots.

The physical chromosome position of the *NADH-GOGAT-3B* gene co-localize with Meta QTLs for high protein content [14]. Besides a QTL for grain protein content also has been reported on chromosome 3AL in the same genetic material used in the present paper in the homoeologous position of the *NADH-GOGAT-3B* gene [17]. In conclusion, our results support the proposition that *NADH-GOGAT* may be a major candidate gene driving NUE on chromosome group 3 in bread and durum wheat. Future experiments might address this proposition via specific absence or overexpression of the NADH-GOGAT genes. Among the potential options for the former is transgene mediated silencing, deletion lines such as used in the present report [31], or use of wheat tilling populations to identify mutations [32]. Gene Overexpression could be accomplished by transformation [33] with gene NADH-GOGAT gene constructs. These strategies are now available with the gene sequences reported in the current report.

## Materials and Methods

### Plant Material and DNA Analysis

The durum wheat cultivars Ciccio and Svevo were used for *NADH-GOGAT* genomic sequence determination. These cultivars are the parentals of a mapping population represented by a set of 120 recombinant inbred lines (RILs) as previously described [22]. The two parents were chosen for differences in important qualitative and quantitative traits; i.e., grain yield components, grain protein content, yellow pigment, and adaptive traits.

Nulli-tetrasomic lines (NTs) of *Triticum aestivum* cv Chinese Spring [23], [24] were used to physically localize *NADH-GOGAT* markers to specific chromosomes. Chinese Spring di-telosomic lines [25] were used for further assignment of markers to each chromosomal arm. Physical location on chromosome bins of each PCR fragment was obtained using a set of eight common wheat deletions lines dividing the 3A and 3B genome chromosomes into bins [26].

Genomic DNA was isolated from fresh leaves using the method as described [27] and subsequently purified by phenol-chloroform extraction. DNA amplifications were carried out as described previously [22]. For qRT-PCR, leaf and root samples were collected from 5 plants of both cultivars in different phenological stages (first leaf, flowering, grain filling).

### Gene Sequence Determination for Chinese Spring and *Triticum tauschii*

To isolate the complete sequences of the three *NADH-GOGAT* genes in hexaploid wheat, we used a reported rice genomic sequence of *NADH*-*GOGATI* [15] as the initial query. The rice sequence was used to probe public databases to extract the 454 sequences of the hexaploid wheat cv Chinese Spring (cerealsdb.uk.net/search_reads.htm) and the 454 reads of *Triticum tauschii* (avena.pw.usda.gov/RHmapping/blast/). These two groups of 454 reads were separately assembled using the SeqMan software by DNAStar (Lasergene). Using the public database at NCBI (http://www.ncbi.nlm.nih.gov/) we also identified a *GOGAT* gene from the wheat B-genome within the previously reported wheat genomic DNA clone FN564429.

### Gene Characterization in the Italian Durum Wheat cvs Ciccio and Svevo

A set of genome specific primer pairs were designed using OligoExplorer and Primer3 (frodo.wi.mit.edu/primer3/) software for the three distinct cv Chinese Spring *GOGAT* hexaploid sequences previously obtained. Single PCR fragments were cloned from cultivar Chinese Spring using the vector pCR4-TOPO and transformed into DH10B electroMAX cells (Invitrogen, Cloning Kit) following the manufacturer’s instructions, and then sequenced at both insert ends with T3 and T7 primers and BigDye chemistry (Applied Biosystems) using a 96 capillary automatic sequencer ABI PRISM 3730XL. Gaps and uncertain sequence were resolved by primer walking. Regions of less coverage or ambiguous reads were rechecked with additional primers.

### Analysis of Sequences

NCBI (ncbi.nlm.nih.gov) was used for annotation of the new wheat BAC sequence by BLAST analyses and total EST analyses by direct querying to NCBI. Exon/intron junctions are predicted by alignment with Triticeae EST sequences, when available, or with other monocot EST if no Triticeae ESTs covered those sequences.

DNA sequences were compared using the Megalign (alignments and dot blots) and Seqman (assemblies) modules of the Lasergene suite (DNAStar, Inc.).

Sources of genomic sequences were as follows: *Arabidopsis thaliana NADH-GOGAT*, Genbank ncbi.nlm.nih.gov (AT5G53460.1); *Brachypodium distachyon*, http://brachypodium.org, (Bradi2g46670.1); poplar (*Populus trichocarpa*), genome.jgi-psf.org (POPTR_0015s01950.1); grape (*Vitis vinifera*), genoscope.cns.fr/externe/GenomeBrowser/Vitis/(GSVIVT01038714001); rice (*Oryza sativa*), gramene.org (LOC_Os01g48960.1); maize (*Zea mays*), http://plantgdb.org/ZmGDB (GRMZM2G077054).

For ease of reading, it will be understand that common names and genus names will be used unless referring to different species than noted above; e.g., *Brachypodium* instead of *B. distachyon* and rice instead of *O. sativa*. Plant ESTs were searched at Genbank, except for *Brachypodium* ESTs that were found at brachypodium.org.

### RNA Isolation for Quantitative Real-time PCR

Tissues of each sample were harvested in each phase, frozen in liquid nitrogen and stored at -80°C until RNA extraction. Total RNA was extracted with *RNeasy Plant Mini Kit* (QIAGEN®) and checked on 1.5% denaturing agarose gels. The total amount of RNA and its purity was determined using Nano-Drop ND1000 spectrophotometer (Thermo Scientific, Walthman, MA, USA). All RNA samples were adjusted to the same concentration (1 µg) for subsequent treatment with *DNase I recombinant* (Roche Applied Science, Mannheim, Germany) in order to remove genomic DNA, and then were reverse-transcribed into double stranded cDNA with *Trascriptor First Strand cDNA Synthesis Kit* (Roche Applied Science, Mannheim, Germany).

### Reference Genes and Specific Gene Primer Design

Genes coding for Cell Division Control AAA-Superfamily of ATPases (*CDC*), ADP-Ribosylation Factor (*ADP-RF*) and RNase L Inhibitor-like protein (*RLI*) were used as reference genes as suggested as novel and stable references for wheat [28], [29] (Table 1). These gene were chosen as they are considered to be stable reference genes (with a stability value around 0.035 for all the three of them) evaluated with NormFinder software [30].

**Table 1:** Primer pairs sequences for housekeeping genes [28].

Gene name	Orientation	Primer 5′-3′ sequence	Product length (bp)	Melt peak (°C)
*CDC*	Forward	CAAATACGCCATCAGGGAGAACATC	227	86.5
	Reverse	CGCTGCCGAAACCACGAGAC		
*RLI*	Forward	CGATTCAGAGCAGCGTATTGTTG	242	80
	Reverse	AGTTGGTCGGGTCTCTTCTAAATG		
*ADP-RF*	Forward	GACCACCATCCTCTACAAG	276	84.5
	Reverse	AGCAGCACAGCATCAC		

DNA sequences of *NADH-GOGAT-3A* and *NADH-GOGAT-3B* from the two durum cvs Svevo and Ciccio were aligned with ClustalW (ebi.ac.uk) to highlight dissimilarities between the two homoeologous genes. Specific primer pairs (Table 2) were designed for each gene in a region of the first exon containing a 3 bp indel using Oligo Explorer software (Gene Link™).

**Table 2:** Primer pairs specific for the two isoforms of the *NADH-GOGAT* gene.

Gene name	Orientation	Primer 5′-3′ sequence	Product length (bp)	Melt peak (°C)
*NADH-GOGAT-A*	Forward	GCGCCATGTCTCTGGAGGGC	113	86
	Reverse	GGGCCGTATCGACTCTGCGT		
*NADH-GOGAT-B*	Forward	CACGGCGCCATGTCCCTGCAT	108	83
	Reverse	GCCTGATGGACTCGGCGTCG		

### qReal-time PCR Reactions

Real-time quantitative PCR was carried out using EVA® GREEN in the CFX96™ Real-time PCR Systems (BIO-RAD). The PCR condition were 95°C for 3 min followed by 40 cycles of 95°C for 10 sec at 60°C for 30 sec. Amplification efficiency (98% to 100%) for the two primer sets was determined by amplification of cDNA with a series of 6 scalar dilution (1∶5) per reaction. In each PCR experiment, 1 µl of a 1∶5 dilution cDNA was used in a final volume of 10 µl containing 5 µl of EvaGreen Mix 10X (Bio-Rad) and 500 nM of each primer. All experiments were performed in Hard-Shell 96-well skirted PCR plates (HSP9601) with Microseal® ‘B’ Adhesive Seals (MSB-1001) from Bio-Rad. Fluorescence signals were collected at each polymerization step.

The specificity of the amplicons was confirmed by the presence of a single band of expected size for each primer pair in agarose gels (2% w/v), by single peak melting curves of the PCR products, and by sequencing of the amplified fragments (BMR Genomics, Padova, Italy).

qRT-PCR data for both *NADH-GOGAT* and endogenous controls genes reported in this paper derive from the mean values of three independent amplification reactions carried out on five different plants harvested in the same phenotypic stage. All calculations and analyses were preformed using CFX Manager 2.1 software (Bio-Rad Laboratories) using the 2^−ΔΔCt^ method which uses the Relative Quantity (RQ) calculated on ratio from relative quantity of the target gene and the relative expression of the reference gene (that includes the three reference targets in each sample). Standard deviation was used to normalize the expression values for the highest or lowest individual expression levels (CFX Manager 2.1 software user manual). All the results were analyzed by ANOVA.

## Supporting Information

Figure S1 **Alignment of 3A, 3B, and 3D** ***NADH-GOGAT*** **gene sequences.** The three cv Chinese Spring *NADH-GOGAT* gene sequences are aligned from 5′ to 3′ regions common to all three genes using the Megalign module of Lasergene (DNAstar, Inc.). Red brackets enclose exons whose numbers are in blue above the alignment. Introns are numbered according to the preceding exon. Intron 13 is indicated in red above the intron. Start and stop codons are boxed in red.
(PDF)

Figure S2 **Comparison of 3A and 3B** ***NADH-GOGAT*** **genes.** The *NADH-GOGAT* genes from the A- and B- genomes were analyzed by dot plot at a criterion of 100% match over a 20 base window.
(TIF)

File S1 **Durum wheat NADH-GOGAT gene sequences.** Fasta sequences of hexaploid cv Chinese Spring *NADH-GOGAT* genes and encoded polypeptides.
(TXT)

## References

[1] Krapp A, Saliba-Colombani V, Daniel-Vedele F (2005) Analysis of C and N metabolisms and of C/N interactions using quantitative genetics. Photosyn Res 83: 251–263 doi:10.1007/s11120-004-3196-7 16143855 10.1007/s11120-004-3196-7

[2] Laperche A, Brancourt-Hulmel M, Heumez E, Gardet O, Le Gouis J (2006) Estimation of genetic parameters of a DH wheat population grown at different N stress levels characterized by probe genotypes. Theor Appl Genet 112: 797–807 doi:10.1007/s00122-005-0176-z 16432739 10.1007/s00122-005-0176-z

[3] Hirel B, Le Gouis J, Ney B, Gallais A (2007) The challenge of improving nitrogen use efficiency in crop plants: towards a more central role for genetic variability and quantitative genetics within integrated approaches. J Exp Bot 58: 2369–2387 doi:10.1093/jxb/erm097 17556767 10.1093/jxb/erm097

[4] Cren M, Hirel B (1999) Glutamine Synthetase in Higher Plants Regulation of Gene and Protein Expression from the Organ to the Cell. Plant Cell Physiol 40: 1187–1193.

[5] Ireland RJ, Lea PJ (1999) The enzymes of glutamine, glutamate, asparagines and aspartate metabolisms. Plant amino acids: Biochemistry and biotechnology. New York: Marcel Dekker. 49–109.

[6] Tobin AK, Yamaya T (2001) Cellular compartmentation of ammonium assimilation in rice and barley. J Exp Bot 52: 591–604. 11373307

[7] Kamachi K, Yamaya T, Mae T, Ojima K (1991) A Role for Glutamine Synthetase in the Remobilization of Leaf Nitrogen during Natural Senescence in Rice Leaves. Plant Physiol 96: 411–417. 16668201 10.1104/pp.96.2.411 PMC1080785

[8] Finnemann J, Schjoerring JK (2000) Post-translational regulation of cytosolic glutamine synthetase by reversible phosphorylation and 14-3-3 protein interaction. Plant J 24: 171–181. 11069692 10.1046/j.1365-313x.2000.00863.x

[9] Kichey T, Hirel B, Heumez E, Dubois F, Le Gouis J (2007) In winter wheat (*Triticum aestivum* L.), post-anthesis nitrogen uptake and remobilisation to the grain correlates with agronomic traits and nitrogen physiological markers. Field Crops Res 102: 22–32.

[10] Chen F-L, Cullimore JV (1989) Location of two isoenzymes of NADH-dependent glutamate synthase in root nodules of *Phaseolus vulgaris* L. Planta. 179: 441–447 doi:10.1007/BF00397583 10.1007/BF00397583 24201767

[11] Baron A, Tobin A, Wallsgrove RM (1994) The kinetics of azaserine and phosphinothricin inhibition of glutamate synthase cycle enzymes from barley leaves. Plant Physiol Biochem 32: 555–560.

[12] Yamaya T, Obara M, Nakajima H, Sasaki S, Hayakawa T, et al (2002) Genetic manipulation and quantitative-trait loci mapping for nitrogen recycling in rice. J Exp Bot 53: 917–925. 11912234 10.1093/jexbot/53.370.917

[13] Hayakawa T, Nakamura T, Hattori F, Mae T, Ojima K, et al (1994) Cellular localization of NADH-dependent glutamate-synthase protein in vascular bundles of unexpanded leaf blades and young grains of rice plants. Planta 193: 455–460 doi:10.1007/BF00201826

[14] Quraishi UM, Abrouk M, Murat F, Pont C, Foucrier S, et al (2011) Cross-genome map based dissection of a nitrogen use efficiency ortho-metaQTL in bread wheat unravels concerted cereal genome evolution. Plant J 65: 745–756 doi:10.1111/j.1365-313X.2010.04461.x 21251102 10.1111/j.1365-313X.2010.04461.x

[15] Goto S, Akagawa T, Kojima S, Hayakawa T, Yamaya T (1998) Organization and structure of NADH-dependent glutamate synthase gene from rice plants. Biochim Biophys Acta 1387: 298–308. 9748637 10.1016/s0167-4838(98)00142-3

[16] Van den Heuvel RHH, Ferrari D, Bossi RT, Ravasio S, Curti B, et al (2002) Structural studies on the synchronization of catalytic centers in glutamate synthase. J Biol Chem 277: 24579–24583 doi:10.1074/jbc.M202541200 11967268 10.1074/jbc.M202541200

[17] Blanco A, Mangini G, Giancaspro A, Giove S, Colasuonno P, et al (2012) Relationships between grain protein content and grain yield components through quantitative trait locus analyses in a recombinant inbred line population derived from two elite durum wheat cultivars. Mol Breeding 30: 79–92 doi:10.1007/s11032-011-9600-z

[18] Anderson OD, Coleman-Derr D, Gu YQ, Heath S (2010) Structural and transcriptional analysis of plant genes encoding the bifunctional lysine ketoglutarate reductase saccharopine dehydrogenase enzyme. BMC Plant Biol 10: 113 doi:10.1186/1471-2229-10-113 20565711 10.1186/1471-2229-10-113 PMC3017810

[19] Vogel JP, Garvin DF, Mockler TC, Schmutz J, Rokhsar D, et al (2010) Genome sequencing and analysis of the model grass *Brachypodium distachyon* . Nature 463: 763–768 doi:10.1038/nature08747 20148030 10.1038/nature08747

[20] Lu Y, Luo F, Yang M, Li X, Lian X (2011) Suppression of glutamate synthase genes significantly affects carbon and nitrogen metabolism in rice (*Oryza sativa* L.). Sci China Life Sci 54: 651–663 doi:10.1007/s11427-011-4191-9 21748588 10.1007/s11427-011-4191-9

[21] Lancien M, Martin M, Hsieh M-H, Leustek T, Goodman H, et al (2002) *Arabidopsis* glt1-T mutant defines a role for NADH-GOGAT in the non-photorespiratory ammonium assimilatory pathway. Plant J 29: 347–358. 11844111 10.1046/j.1365-313x.2002.01218.x

[22] Gadaleta A, Giancaspro A, Giove SL, Zacheo S, Mangini G, et al (2009) Genetic and physical mapping of new EST-derived SSRs on the A and B genome chromosomes of wheat. Theor Appl Genet 118: 1015–1025 doi:10.1007/s00122-008-0958-1 19183861 10.1007/s00122-008-0958-1

[23] Sears ER (1954) The aneuploids of common wheat. University of Missouri, College of Agriculture, Agricultural Experiment Station Bulletin 572: 1–58.

[24] Sears ER (1966) Nullisomic-tetrasomic combinations in hexaploid wheat. Chromosome manipulations and plant genetics. Edinburgh: Oliver and Boyd.

[25] Sears ER, Sears LMS (1978) The telocentric chomosomes of common wheat. Proceedings of the 5th International wheat genetics symposium. New Delhi: Indian Society of Genetics and Plant Breeding. 389–407.

[26] Endo TR, Gill BS (1996) The Deletion Stocks of Common Wheat. J Hered 87: 295–307.

[27] Sharp PJ, Kreis M, Shewry PR, Gale MD (1988) Location of β-amylase sequences in wheat and its relatives. Theoret Appl Genetics 75: 286–290 doi:10.1007/BF00303966

[28] Paolacci AR, Tanzarella OA, Porceddu E, Ciaffi M (2009) Identification and validation of reference genes for quantitative RT-PCR normalization in wheat. BMC Mol Biol 10: 11 doi:10.1186/1471-2199-10-11 19232096 10.1186/1471-2199-10-11 PMC2667184

[29] Giménez MJ, Pistón F, Atienza SG (2011) Identification of suitable reference genes for normalization of qPCR data in comparative transcriptomics analyses in the Triticeae. Planta 233: 163–173 doi:10.1007/s00425-010-1290-y 20960006 10.1007/s00425-010-1290-y

[30] Andersen CL, Jensen JL, Ørntoft TF (2004) Normalization of Real-Time Quantitative Reverse Transcription-PCR Data: A Model-Based Variance Estimation Approach to Identify Genes Suited for Normalization, Applied to Bladder and Colon Cancer Data Sets. Cancer Res 64: 5245–5250 doi:10.1158/0008-5472.CAN-04-0496 15289330 10.1158/0008-5472.CAN-04-0496

[31] Qi L, Echalier B, Friebe B, Gill BS (2003) Molecular characterization of a set of wheat deletion stocks for use in chromosome bin mapping of ESTs. Funct Integr Genomics 3: 39–55. 12590342 10.1007/s10142-002-0063-5

[32] Chen L, Huang L, Min D, Phillips A, Wang S, et al (2012) Development and Characterization of a New TILLING Population of Common Bread Wheat (*Triticum aestivum* L.) PLoS One. 7: e41570. 10.1371/journal.pone.0041570 PMC3402408 22844501

[33] Gadaleta A, Blechl AE, Nguyen S, Cardone MF, Ventura M, et al.. (2008) Stably expressed D-genome-derived HMW glutenin subunit genes transformed into different durum wheat genotypes change dough mixing properties. Mol Breeding 22: 267–279. doi 10.1007/s11032-008-9172-8.

