# Supplementary figures and images for: Structural Analysis of the Wheat Genes Encoding NADH-Dependent Glutamine-2-oxoglutarate Amidotransferases and Correlation with Grain Protein Content
Source: PLoS One. 2013 Sep 17;8(9):e73751. doi: 10.1371/journal.pone.0073751 (PMC3775782; doi:10.1371/journal.pone.0073751)

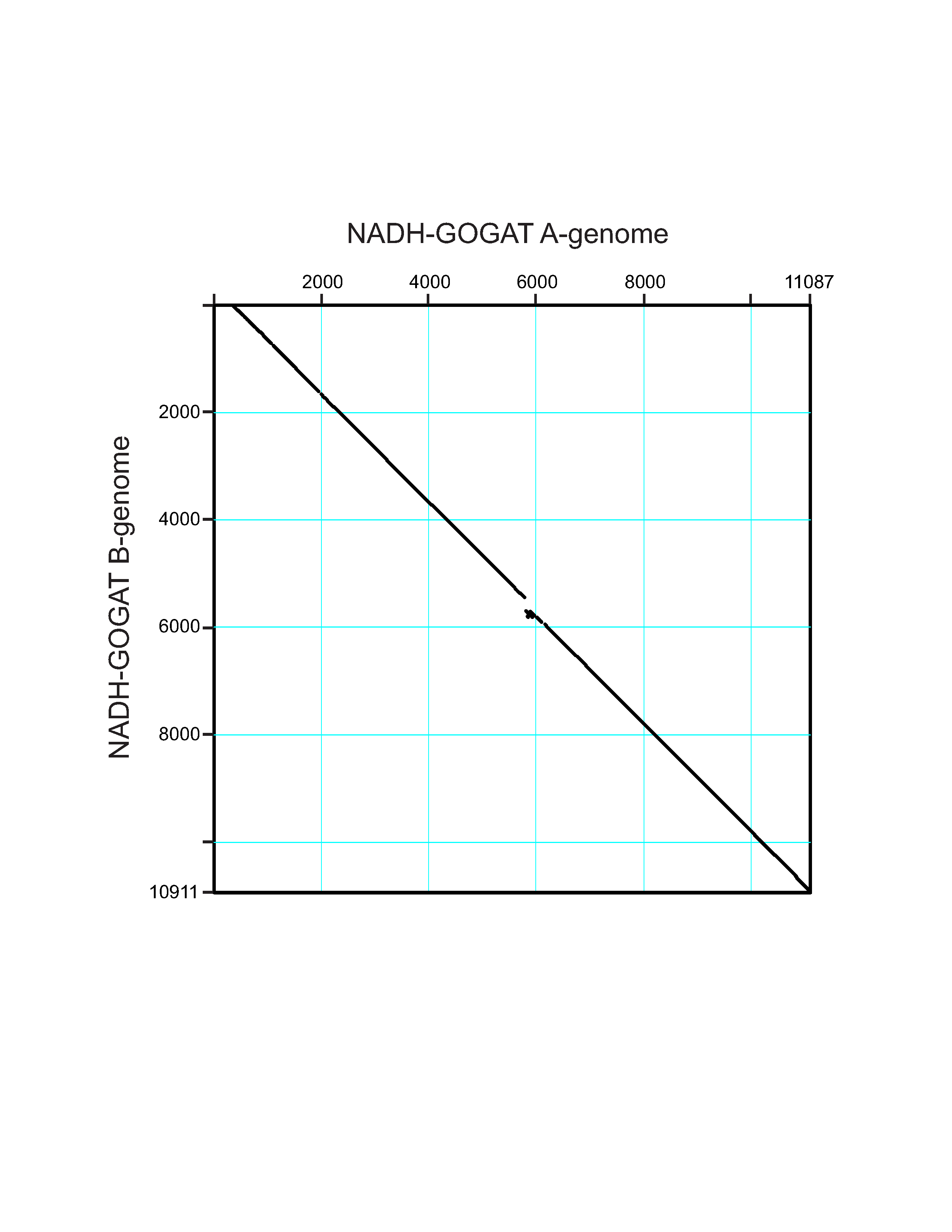

Supplement: Figure S2 — Comparison of 3A and 3B NADH-GOGAT genes. The NADH-GOGAT genes from the A- and B- genomes were analyzed by dot plot at a criterion of 100% match over a 20 base window. (TIF) [file pone.0073751.s002.tif]
