# Supplementary material for: Structural Analysis of the Wheat Genes Encoding NADH-Dependent Glutamine-2-oxoglutarate Amidotransferases and Correlation with Grain Protein Content
Source: PLoS One. 2013 Sep 17;8(9):e73751. doi: 10.1371/journal.pone.0073751 (PMC3775782; doi:10.1371/journal.pone.0073751)

Alignment Report of Untitled ClustalV (Weighted)
Wednesday, March 20, 2013 1:05 PM

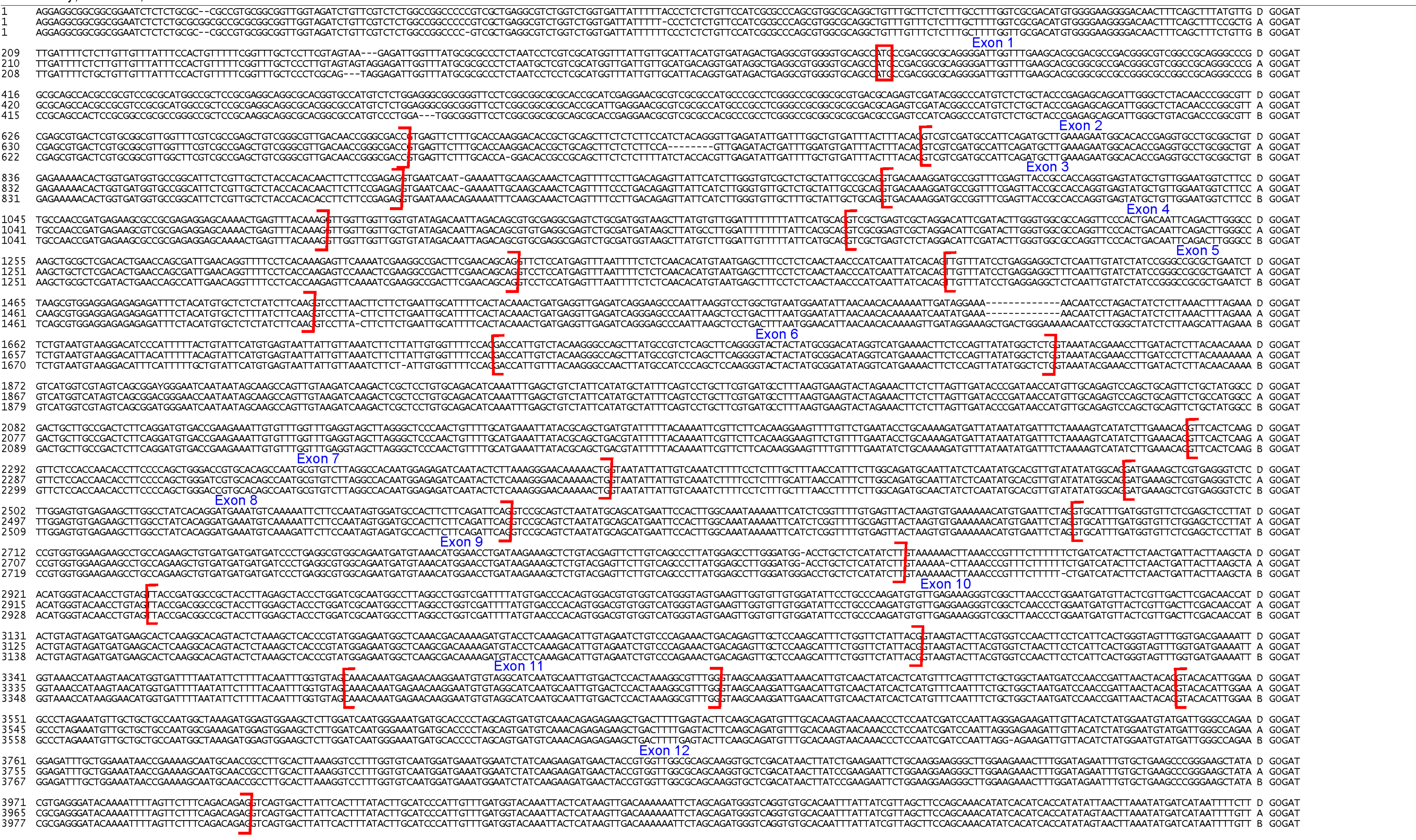

Alignment Report of Untitled ClustalV (Weighted)
Wednesday, March 20, 2013 1:05 PM

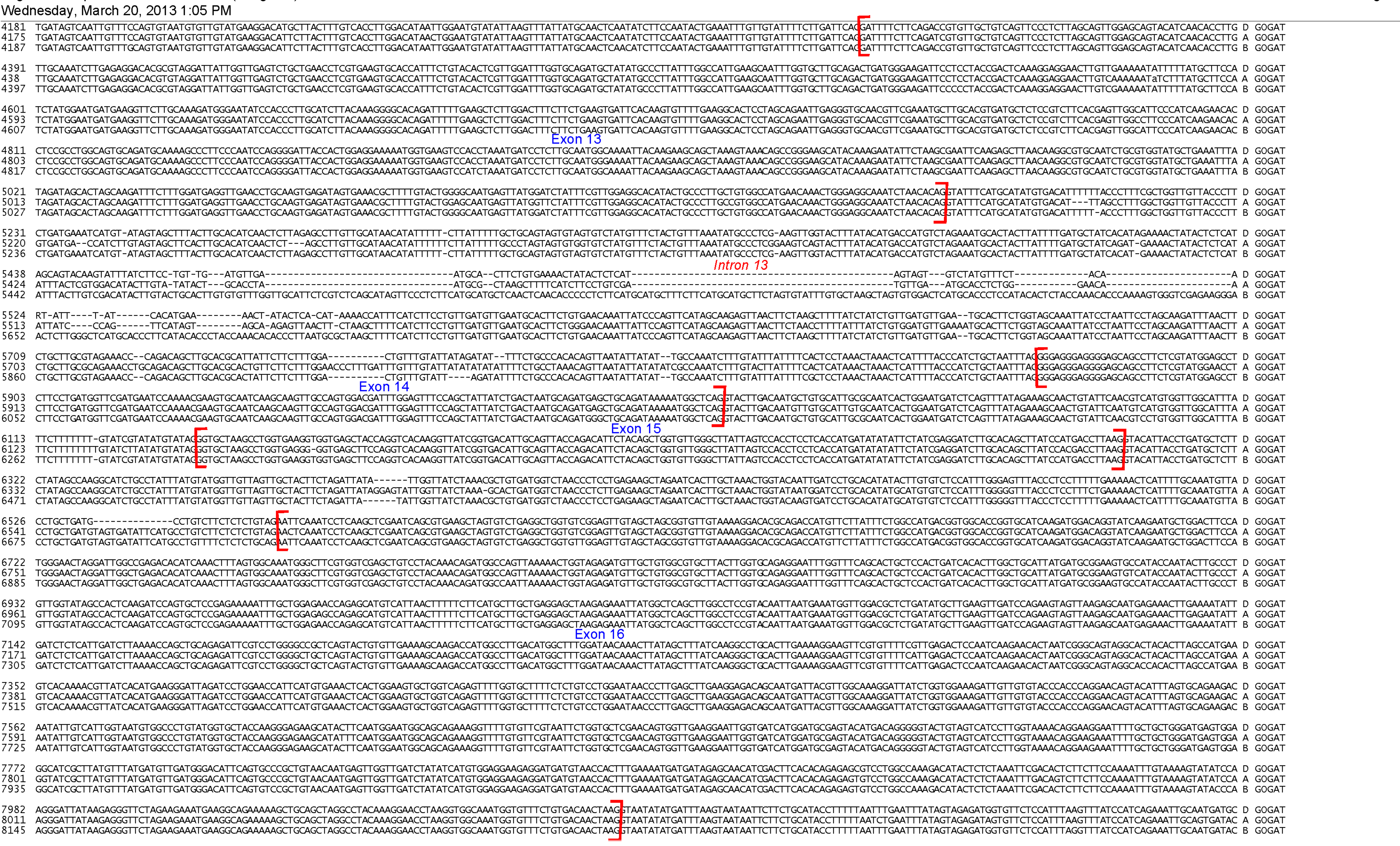

Alignment Report of Untitled ClustalV (Weighted)
Wednesday, March 20, 2013 1:05 PM

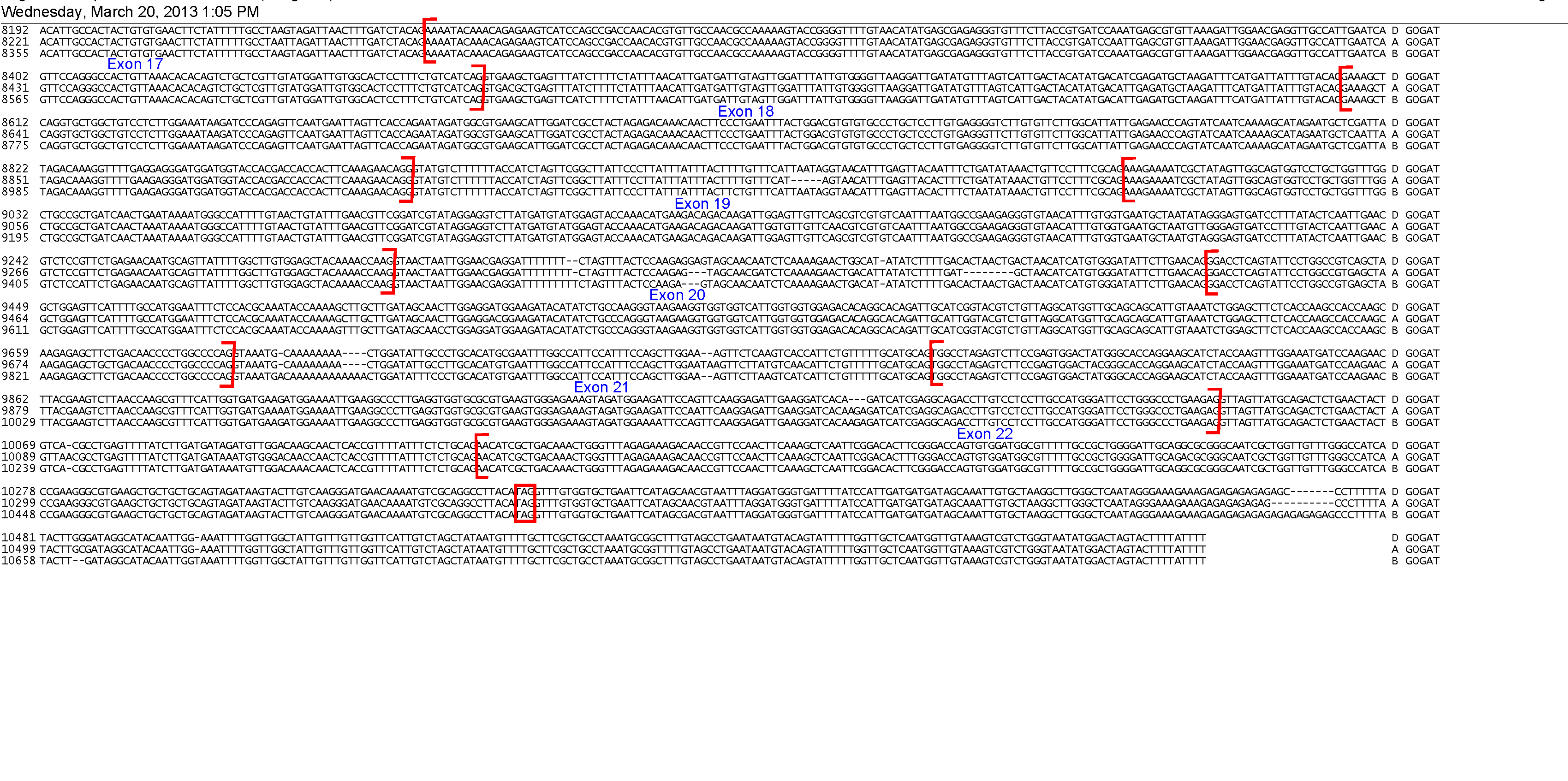

Supplement: Figure S1 — Alignment of 3A, 3B, and 3D NADH-GOGAT gene sequences. The three cv Chinese Spring NADH-GOGAT gene sequences are aligned from 5′ to 3′ regions common to all three genes using the Megalign module of Lasergene (DNAstar, Inc.). Red brackets enclose exons whose numbers are in blue above the alignment. Introns are numbered according to the preceding exon. Intron 13 is indicated in red above the intron. Start and stop codons are boxed in red. (PDF) [file pone.0073751.s001.pdf]
